# The Importance of Poisoning vs. Road Traffic Injuries as a Cause of Death in Rural Sri Lanka

**DOI:** 10.1371/journal.pone.0000599

**Published:** 2007-07-11

**Authors:** Michael Eddleston, Nilantha Udayakumara, Sriyantha Adhikari, Dhamika de Silva, M. H. Rezvi Sheriff, Dhananjaya L. Waidyaratne

**Affiliations:** 1 Centre for Tropical Medicine, Nuffield Department of Clinical Medicine, University of Oxford, Oxford, United Kingdom; 2 Ox-Col Collaboration, Department of Clinical Medicine, University of Colombo, Colombo, Sri Lanka; 3 South Asian Clinical Toxicology Research Collaboration, Sri Lanka; 4 Office of the Provincial Director of Health Services, Anuradhapura, North Central Province, Sri Lanka; 5 Office of the Judicial Medical Officer, Anuradhapura General Hospital, North Central Province, Sri Lanka; The Canberra Hospital, Australia

## Abstract

**Background:**

Road traffic crashes are considered by the WHO to be the most important global cause of death from injury. However, this may not be true for large areas of rural Asia where road vehicles are uncommon. The issue is important, since emphasising the importance of road traffic crashes risks switching resources to urban areas, away from already underfunded rural regions. In this study, we compared the importance of road traffic crashes with other forms of injury in a poor rural region of South Asia.

**Methodology/Principal Findings:**

We collected data on all deaths from injury in the North Central Province of Sri Lanka (NCP; population 1,105,198 at 2001 census) over 18 months using coronial, hospital, and police data. We calculated the incidence of death from all forms of intentional and unintentional injury in the province. The annual incidence of death from injury in the province was high: 84.2 per 100,000 population. Half of the deaths were from self-harm (41.3/100,000). Poisoning (35.7/100,000)—in particular, pesticide self-poisoning (23.7/100,000)—was the most common cause of death, being 3.9-fold more common than road traffic crashes (9.1/100,000).

**Conclusions/Significance:**

In poor rural regions of South Asia, fatal self-harm and pesticide self-poisoning in particular are significantly more important than road traffic injuries as a cause of death. It is possible that the data used by the WHO to calculate global injury estimates are biased towards urban areas with better data collection but little pesticide poisoning. More studies are required to inform a debate about the importance of different forms of injury and how avoidable deaths from any cause can be prevented. In the meantime, marked improvements in the effectiveness of therapy for pesticide poisoning, safer storage, reduced pesticide use, or reductions in pesticide toxicity are required urgently to reduce the number of deaths from self-poisoning in rural Asia.

## Introduction

Injuries are estimated to cause around 5.8 million deaths each year worldwide, 12% of all deaths [1]. Road traffic injuries are thought to be the most common cause of death, resulting in 1.26 million each year (20.7% of all deaths from injury) [2]. The majority of the remaining 4.6 million deaths occur from self-inflicted injuries, drowning, violence, and poisoning [2], [3].

Road traffic crashes have recently risen to prominence in global public health policy [4]. The WHO dedicated the World Health Day in 2004 to road safety and together with the World Bank published a report in 2004 entitled the *World Report on Road Traffic Injury Prevention* [5]. The WHO's Department of Injuries and Violence Prevention was established in 2001 and concentrates much of its resources on road traffic crashes.

However, road traffic crashes are likely to be most important in urban areas where the mix of vehicles and pedestrians is greatest [4]. In poor rural areas of the developing world, there are relatively few vehicles or roads and other forms of injury may be more important. Health care provision is already worse in rural compared to urban areas of the Asian developing world [6], [7]. If road traffic crashes are not as important in poor rural areas, expanding resources in response to road traffic crashes at the expense of other injuries risks increasing the already gross inequalities in health care provision between rural and urban populations.

As part of our ongoing studies in Sri Lanka, we determined the importance of road traffic injuries in a poor rural region compared to other forms of injury.

## Methods

**Figure 1:**
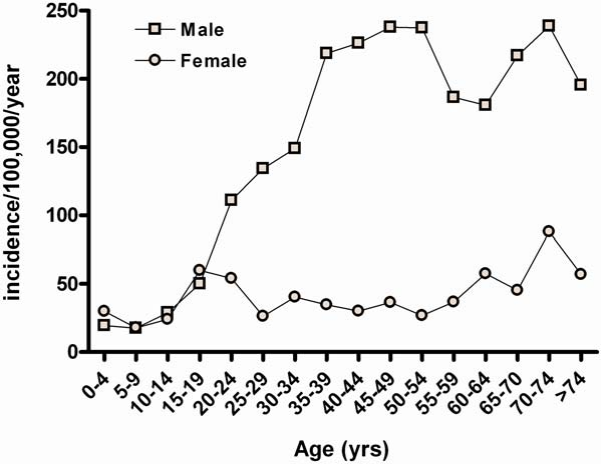
Incidence of fatal injury by gender and age. The incidence was calculated from the gender and age specific 2001 census results. The Government of Sri Lanka estimates that the population of the province has increased by 1.2% each year since the census, reducing the incidences shown here.

This study was based around a prospective study of poisoned patients presenting to secondary referral hospitals in the North Central Province (NCP) of Sri Lanka that started in March 2002. The province is predominantly rural and had a population of 1,105,198 at the 2001 census and an estimated mid-year population of 1,132,000 in 2003 (estimated 1.2% annual increase in population since the census; male 571,735 female 560,320).[8] Incidences were calculated using the 2003 estimated mid-year population, except for age specific incidences used in figure 1.

All deaths in Sri Lanka must be reported to the police. Deaths from injury are recorded in separate registers for sudden deaths, accidents, suicides, and grave crimes. Post-mortems are carried out on all injury deaths by Judicial Medical Officers (JMO) in district hospitals or District Medical Officers in smaller peripheral hospitals. Coroner's inquiries are then held to determine cause of death and intent and the report filed with the local Magistrate's Court; receipt of the report by the court results in the Coroner being paid. People dying outside of NCP, after transfer to a tertiary hospital outside of the Province, will be present in NCP police registers but not NCP coroners' reports.

Starting in May 2004, details on all deaths from injury occurring in the province from January 1, 2003, until June 30, 2004, were manually extracted from JMOs' records in the secondary district hospitals, from the coroners' official records stored in all six Magistrate's Courts, from the registers in all 30 police stations, and the Ox-Col prospective study of poisoning deaths in the district hospitals. The study was carried out with the full knowledge and support of the Provincial Director of Health Services, Senior Superintendent of Police, and Chief Magistrate. Ethics approval was obtained from the Faculty of Medicine Research Ethics Committee, University of Colombo, and Oxford Tropical Research Ethics Committee.

The data was entered into an access database using a Visual basic interface. The database was then reviewed by two people independently to identify multiple entries for a single death and a single entry resolved. Simple descriptive statistics were used to describe deaths according to gender, cause of death, and place of death.

## Results

3656 records of injury deaths were recorded from the multiple sources. Review of these entries identified 1430 deaths in 18 months. 989 deaths were noted in multiple sources (69.2%; mean number of records 2.97) while 441 had a single entry. 260 of the 441 deaths with single entries (59.0%) were from police records, while the remaining records came from Magistrate and Hospital records.

The incidence of death from injury in the Province was 84.2 per 100,000 population per year. Deaths was more common in men (1121; incidence 130.7/100,000) than women (309; incidence 36.8/100,000; table 1). The incidence was similar for both males and females under 20 years (figure 1; around 20/100,000 in those under 15 and 50–60/100,000 in 15–19 year olds); thereafter, the incidence rose sharply for males peaking at 238/100,000 in 45–54 year olds and again in 70–74 year olds. In contrast, fatality rates for women remained steady between 25 and 85/100,000.

**Table 1:** Deaths from injury in North Central Province by cause, age, and gender during the 18 months, Jan 2003–Jun 2004.

	Total (%)	Male adult	Male child <15 years	Female adult	Female child <15 years
**Animal attack**	109 (7.6)	67	13	19	10
* Bull*	*2*	*1*	*0*	*1*	*0*
* Dog*	*5*	*4*	*1*	*0*	*0*
* Elephant*	*41*	*35*	*2*	*4*	*0*
* Snake bite*	*60*	*26*	*10*	*15*	*9*
* Wasp sting*	*1*	*1*	*0*	*0*	*0*
**Assault**	146 (10.2)	115	10	15	6
* Blunt weapon*	*33*	*27*	*2*	*4*	*0*
* Blast injuries/gun shot*	*50*	*37*	*7*	*3*	*3*
* Stab injury*	*51*	*45*	*0*	*6*	*0*
* Strangled*	*12*	*6*	*1*	*3*	*2*
**Burns**	59 (4.1)	22	0	33	4
**Drowning**	135 (9.4)	91	10	22	12
**Electrocution**	25 (1.7)	21	1	3	0
**Falls**	49 (3.4)	44	0	2	3
**Falling objects**	8 (0.6)	5	1	1	1
**Hanging**	93 (6.5)	85	0	8	0
**Hit by train**	31 (2.2)	29	0	2	0
**Lightning**	10 (0.7)	6	1	3	0
**Road traffic crash**	154 (10.8)	124	9	15	6
**Poisoning**	606 (42.4)	460	4	130	12
Pesticides	*403*	*324*	*0*	*75*	*4*
Plants	*81*	*48*	*1*	*29*	*3*
**Illegal abortion**	1 (0.1)	0	0	1	0
**Unknown**	3 (0.3)	2	0	1	0
**Total**	**1430**	**1071**	**50**	**255**	**54**

The commonest mode of death was poisoning, and in particular pesticide poisoning, which caused 606 and 403 deaths, respectively (table 1), giving incidences of 35.7 and 23.7/100,000 per year. Fatal self-poisoning and pesticide self-poisoning were 3.2 and 4.0 times more common in men than women. Self-poisoning with seeds of the yellow oleander tree (*Thevetia peruviana*) was the other common form of fatal poisoning, causing 81 deaths (incidence 4.8/100,000) but with a more equal male to female ratio (table 1).

Road traffic injuries were the next most common cause of death, but were responsible for only 10.8% of deaths (incidence 9.1/100,000), 3.9-fold less than poisoning. The other forms of common injury death: assault, drowning, and hanging, caused 10.2%, 9.4%, and 6.5% of deaths, respectively (table 1). Interestingly, animal attacks were responsible for a relatively large number of deaths (109, 7.6%). Snake bite killed 60 people (4.2%) while elephants killed 41 (2.9%). Elephants also indirectly caused most of the 25 deaths due to electrocution since the men were manipulating high-power electricity lines around their fields in an attempt to build fences to fend off marauding elephants.

The form of intent for most death was determined by Coroners' enquiries (table 2). Intentional self-injury was the most common mode of death (49.0%, 41.3/100,000 per year). Unintentional injuries were responsible for 34.1% (28.7/100,000) and violence 7.6% (6.4/100,000) of deaths. The form of intent could not be determined for 133 deaths (9.3%).

**Table 2:** Intent for different methods of death from injury.

	Male (number)				Female (number)			
	Self-harm	Violence	Unintentional	Unknown	Self-harm	Violence	Unintentional	Unknown
**Animal attack**	0	0	80	0	0	0	29	0
**Assault**	8	91*	0	26	0	13*	0	8
**Burns**	5	0	13	5	15	2	17	3
**Drowning**	4	0	83	14	4	0	29	1
**Electrocution**	0	0	21	1	0	0	3	0
**Falling objects**	0	0	6	0	0	0	2	0
**Falls**	1	0	37	6	0	0	5	0
**Hanging**	80	0	1	4	8	0	0	0
**Hit by train**	17	0	6	6	2	0	0	0
**Lightning**	0	0	7	0	0	0	3	0
**Poisoning**	426	2	7	29	130	0	0	12
**Road traffic crash**	1	0	116	16	0	0	19	2

* Assault injuries due to violence include deaths from munitions and roadside bombs, whether exploded intentionally or unintentionally.

Poisoning was responsible for 79.3% of all self-harm deaths, followed by hanging (12.6%), burns (2.9%) and stepping in front of trains (2.7%). Self-harm deaths by drowning were uncommon (1.1%). Although more women than men burned themselves, poisoning was still the overwhelmingly most common method of fatal self-harm in women (table 2).

## Discussion

This study clearly shows the importance of pesticide poisoning in a rural region of the Asian developing world where there are few vehicles and many households using pesticides. Other studies from Tamil Nadu have shown a similar pattern of injuries, with very high rates of pesticide self-harm and low rates of road traffic injury [9]–[11]. A Chinese study suggested that more than 150,000 deaths from pesticides occur every year in China alone [12], [13]. It is therefore likely that pesticide self-poisoning is a major problem across the whole rural Asian developing world [14], [15].

These results contrast with the official WHO estimates for injury mortality in South East Asia (table 3).[16] In the low adult and child mortality stratum SEAR-B (of which Sri Lanka is part), the estimated number of deaths from road traffic crashes is twice the number of deaths from all forms of self-harm. In north central Sri Lanka, the incidence of road traffic crashes was 2.5–3 fold less common (including cases with unknown intention).

**Table 3:** Deaths from injury in the WHO SEAR-B region of South East Asia during 2002 [16] and the North Central Province of Sri Lanka during 2003–4.

Mortality stratum *	SEAR-B Number (%)	SEAR-B Incidence (per 100,000/year)	North Central Province Incidence (per 100,000/year)
**Population**		***298 234 000***	***1 132 000 *** **
**All injuries**	**225 000**	**75.4**	**84.2**
**Unintentional injuries**	**149 000 (66.2)**	**50.0**	**28.7**
Road traffic crashes	72 000 (32.0)	24.1	8.0
Poisoning	8 000 (3.6)	2.7	0.4
Falls	15 000 (6.7)	5.0	2.5
Fires	14 000 (6.2)	4.7	1.8
Drowning	14 000 (6.2)	4.7	6.6
Other unintentional injuries	26 000 (11.6)	8.7	9.3
**Intentional injuries**	**76 000 (33.8)**	**25.5**	**47.7**
Self-inflicted	37 000 (16.4)	12.4	41.3
Violence	28 000 (12.4)	9.4	6.4
War	10 000 (4.4)	3.4	***
**Unknown intent**	-	-	7.8

* Sear-B South-East Asia with low child and low adult mortality [Indonesia, Sri Lanka, Thailand]

** Estimated mid year population for 2003

*** Since a ceasefire was in effect for the civil war during this period, all deaths due to bombs and shootings were included under Violence rather than War.

Globally, great importance has become attached to the large numbers of deaths that result from road traffic injuries in urban areas [4], [17], with new initiatives being started by the WHO and World Bank [5], [17]. However, health care resources are already concentrated in urban areas, to the detriment of health care in poor rural areas. Increasing resources for prevention of road traffic injuries at the expense of other forms of injury risks will increase this disparity further.

It will be important to better define the patterns of death from injuries across rural and urban developing world [18] and to identify effective interventions for all causes of avoidable death. Where public health resources are limited, discussion will be required to decide whether interventions should be selected based on which injuries are most likely to be preventable and/or which intervention is the most cost-effective.

The main limitation of this study is its reliance on retrospective capture of injury deaths from multiple sources. The majority of deaths had undergone a coroner's enquiry which accorded the method of death and intent. We did not independently assess the accuracy of the intent accorded through this enquiry. Our finding that the great majority of patients dying from poisoning in hospital had taken the pesticide intentionally suggests that the intent is recorded reasonably accurately.[19] However, it seems anomalous that 73% of deaths from train injuries were recorded as intentional while only one death from road traffic injury (0.6%) was recorded as intentional. It may also reflect a reluctance to assign suicide as a cause of death without compelling circumstantial evidence. If true, then fatal self-inflicted harm may be being under-reported.

Bearing this caveat in mind, self-harm caused 49% of deaths from injury in this region, compared to 37.3% from unintentional injuries and just 7.6% from violence. The form of intent could not be determined for 9.3% of deaths.

The low figure for violence is surprising since the North Central Province has been heavily involved in the Sri Lankan civil war for the last 25 years. Perhaps the number of homicide deaths was reduced by the ceasefire that begun in late 2001; however, the high number of soldiers and small arms available in the province might be expected to have increased this proportion.

As noted in other parts of Asia, pesticides and hanging are the most common forms of fatal self-harm. However, unlike in rural and semi-urban areas of Tamil Nadu where pesticides and hanging are equally important [9]–[11], pesticides poisoning in North Central Sri Lanka 6-times more important than hanging. This is more similar to China where pesticide poisoning is 3-fold more common than hanging [12]. Since pesticide poisoning is frequently associated with low intent to die [20]–[22], and a period of hours to days before death [23], regulation of the pesticides available, reduced pesticide use in agriculture, and improved medical management will be important measures to reduce this number of deaths.

One surprising feature of this study was the importance of injuries from animals as a cause of death. Over 18 months, elephants were directly or indirectly responsible for the deaths of more than 60 people. Snake bite killed 60 people (3.5/100,000), the majority of patients after contact with health services. Over the last 20 years, with the provision of an Indian polyvalent antivenom to peripheral hospitals, there has been a marked increase in the number of bitten people coming directly to hospital (rather than via a traditional doctor) and in the speed of presentation [24]. As a result, there has been an increase in the number of cases admitted to hospital-from 12,175 in 1991 to 37,081 in 2000-and a fall in the case fatality ratio [25].

The high number of snake bite deaths after hospital admission in NCP is therefore surprising. The reasons may be that patients presented too late for medical care, the antivenom was ineffective, facilities for intubation and ventilation were insufficient (all three most likely with common krait *Bungarus caereleus* bite [24], [26]), or a severe anaphylactoid reaction to the antivenom [27]. Prospective studies are required to determine in detail why deaths still occur from snake bite in Sri Lanka when the majority of bitten individuals survive long enough to reach hospital. The situation seems to be quite different to other parts of Asia where many snake bite deaths occur due to a lack of antivenom and a difficulty in transporting patients to hospital [28]. Better antivenom provision in rural districts, before referral hospitals, needs to become a public health priority in many rural regions [29].

## References

[1] World Health Organization (2001). World Health Report 2001. Mental health: new understanding, new hope..

[2] Krug EG (2004). Injury surveillance is key to preventing injuries.. Lancet.

[3] Krug EG, Dahlberg LL, Mercy JA, Zwi AB, Lozano R (2002). World report on violence and health..

[4] Nantulya VM, Reich MR (2002). The neglected epidemic: road traffic injuries in developing countries.. BMJ.

[5] Peden M, Scurfield R, Sleet D, Mohan D, Hyder AA (2004). World report on road traffic injury prevention..

[6] International Fund for Agriculture Development (2002). Assessment of rural poverty. Asia and the Pacific..

[7] WHO Regional Office for the Western Pacific (2005). Fact Sheet-Health, poverty and MDG..

[8] Department of Census and Statistics GoSL (2007). Mid-Year Population/Vital Statistics by District..

[9] Joseph A, Abraham S, Muliyil JP, George K, Prasad J (2003). Evaluation of suicide rates in rural India using verbal autopsies,1994–9.. BMJ.

[10] Bose A, Konradsen F, John J, Suganthy P, Muliyil J (2006). The mortality rate and years of life lost from unintentional injury and suicide in south India.. Trop Med Int Health.

[11] Gajalakshmi V, Peto R (2007). Suicide rates in rural Tamil Nadu, south India: verbal autopsy of 39 000 deaths in 1997–98.. Int J Epidemiol.

[12] Phillips MR, Yang G, Zhang Y, Wang L, Ji H (2002). Risk factors for suicide in China: a national case-control psychological autopsy study.. Lancet.

[13] Yang GH, Phillips MR, Zhou MG, Wang LJ, Zhang YP (2005). Understanding the unique characteristics of suicide in China: National Psychological Autopsy Study.. Biomed Environ Sci.

[14] Eddleston M, Phillips MR (2004). Self poisoning with pesticides.. BMJ.

[15] Bertolote JM, Fleischmann A, Eddleston M, Gunnell D (2006). Deaths from pesticide poisoning: a global response.. Brit J Psychiat.

[16] World Health Organization (2004). The World Health Report: 2004. Changing history..

[17] Ameratunga S, Hijar M, Norton R (2006). Road-traffic injuries: confronting disparities to address a global-health problem.. Lancet.

[18] Eddleston M, Konradsen F (2007). Time for a re-assessment of the incidence of intentional and unintentional injury in India and South East Asia.. Int J Epidemiol.

[19] Eddleston M, Gunnell D, Karunaratne A, De Silva D, Sheriff MHR (2005). Epidemiology of intentional self-poisoning in rural Sri Lanka.. Brit J Psychiat.

[20] Li XY, Yu YC, Wang YP, Yang RS, Zhang C (2002). Characteristics of serious suicide attempts treated in general hospitals.. Chinese J Mental Health.

[21] Pearson V, Phillips MR, He FS, Ji HY (2002). Attempted suicide among young rural women in the People's Republic of China: possibilities for prevention.. Suicide Life Threat Behav.

[22] Eddleston M, Karunaratne A, Weerakoon M, Kumarasinghe S, Rajapakshe M (2006). Choice of poison for intentional self-poisoning in rural Sri Lanka.. Clin Toxicol.

[23] Eddleston M, Eyer P, Worek F, Mohamed F, Senarathna L (2005). Differences between organophosphorus insecticides in human self-poisoning: a prospective cohort study.. Lancet.

[24] Kularatne SAM (2002). Common krait (*Bungarus caeruleus*) bite in Anuradhapura, Sri Lanka: a prospective clinical study, 1996–98.. Postgrad Med J.

[25] Fernando R (1998). Snake bite.. Management of poisoning.

[26] Theakston RDG, Phillips RE, Warrell DA, Galigedara Y, Abeysekera DTDJ (1990). Envenoming by the common krait (*Bungarus caeruleus*) and Sri Lankan cobra (*Naja naja*): efficacy and complication of therapy with Haffkine antivenom.. Trans R Soc Trop Med Hyg.

[27] Gawarammana IB, Kularatne SAM, Dissanayake WP, Kumarasiri RP, Senanayake N (2004). Parallel infusion of hydrocortisone+/−chlorpheniramine bolus injection to prevent acute adverse reactions to antivenom for snakebites.. Med J Aust.

[28] Sharma SK, Chappuis F, Jha N, Bovier PA, Loutan L (2004). Impact of snake bites and determinants of fatal outcomes in southeastern Nepal.. Am J Trop Med Hyg.

[29] Gutierrez JM, Theakston RDG, Warrell DA (2006). Confronting the neglected problem of snake bit envenoming: the need for a global partnership.. PLOS Medicine.

